# Paeoniflorin Resists H_2_O_2_-Induced Oxidative Stress in Melanocytes by *JNK/Nrf2/HO-1* Pathway

**DOI:** 10.3389/fphar.2020.00536

**Published:** 2020-04-28

**Authors:** Jinping Yuan, Yansong Lu, Hexiao Wang, Yuxin Feng, Shibin Jiang, Xing-Hua Gao, RuiQun Qi, Yan Wu, Hong-Duo Chen

**Affiliations:** ^1^Key Laboratory of Immunodermatology, Ministry of Education, Department of Dermatology, The First Hospital of China Medical University, Shenyang, China; ^2^National and Local Joint Engineering Research Center of Immunodermatological Theranostics, The First Hospital of China Medical University, Shenyang, China

**Keywords:** paeoniflorin, vitiligo, oxidative stress, melanocytes, nuclear factor E2-related factor 2

## Abstract

Paeoniflorin (PF) possesses multiple biological functions including anti-oxidization. PF is the major bioactive ingredient of total glycosides of paeony (TGP), which could promote re-pigmentation of vitiligo. The study was sought to investigate the effects and potential signaling pathways of PF on hydrogen peroxide (H_2_O_2_)-induced oxidative stress in melanocytes. The results showed that pretreatment with 50 µM PF significantly inhibited cell apoptosis, enhanced cell viability, and suppressed reactive oxygen species (ROS) accumulation by enhancing the productions of superoxide dismutase (SOD) and antioxidant enzymes catalase (CAT). Furthermore, PF activated c-Jun amino terminal kinase (*JNK*) and the nuclear factor E2-related factor 2 (*Nrf2*)/heme oxygenase-1 (*HO-1*) pathway to counteract H_2_O_2_-induced oxidative damage in PIG1 and PIG3V. Taken together, our study firstly demonstrates that PF resists H_2_O_2_-induced oxidative stress in melanocytes probably by activating *JNK/Nrf2/HO-1* signaling, suggesting a potential therapeutic application of PF on vitiligo.

## Introduction

Vitiligo is an acquired disorder which characterized by the loss of functional melanocytes in skin and/or mucosa, and affects 0.5% to 2.0% populations all over the world (Ezzedine et al., 2015; Xie et al., 2016). Although the etiology has not been fully established, H_2_O_2_ induced oxidative stress is a key factor during the vitiligo onset and progression (Schallreuter et al., 2007). The pathogenic roles of oxidative damage in vitiligo was reflected in impaired activation of nuclear factor E2-related factor 2-antioxidant response element (*Nrf2-ARE*) pathway, elevated lipid peroxidation, CAT and SOD, etc (Dell'Anna et al., 2007; Liu et al., 2010; Jian et al., 2014; Chang et al., 2017). The components which are involved in oxidative stress pathway increasingly gained the attentions to become potential therapeutic targets in the treatment of vitiligo.

The transcription factor *Nrf2* plays a key role in the expression of phase II antioxidant enzymes which was mediated by antioxidant response element (*ARE*), to prevent cell damage caused by oxidative stress (Zhang et al., 2014). In response to oxidative stress, *Nrf2* can release from Kelch-like ECH-associating protein 1 (Keap1) and translocates to the nucleus and then upregulating the *NQO1* and *HO-1* expression. Decreased *Nrf2* signaling pathway can lead to the decreased ability of melanocytes to prevent oxidative damage in patients with vitiligo (Jian et al., 2011; Jian et al., 2014). So elevated *Nrf2* expression may be helpful in the treatment of vitiligo.

PF is a monoterpene glycoside compound, and extracted from the roots of peony plant (Paeonia lactiflora Pall.) (Wang et al., 2019). PF is the main bioactive ingredient of total glycosides of paeony (TGP) and the latter could promote recovery of depigmentation in patients with vitiligo by increasing CD4^+^/CD8^+^ T lymphocyte ratio and CD4^+^CD25^+^ Treg cell level (Ye et al., 2013; Shen et al., 2019). PF was reported to ameliorate oxidative stress *via* activation of AMP-activated protein kinase (*AMPK*) (Zhu et al., 2018).

Previous studies revealed that oxidative stress is a pathogenic factor of vitiligo, TGP has effects on promoting re-pigmentation of vitiligo lesions, PF can act as a potential antioxidative agent by activating the *Nrf2/HO-1* pathway (Yu et al., 2013) and involved in the melanin synthesis (Hu et al., 2020). Nevertheless, whether TGP or PF can decrease the oxidative damage of melanocytes in vitiligo patients has not yet been studied. In the present study, we examined the effects of PF on H_2_O_2_-induced melanocytes and we may provide potential therapeutic drugs for vitiligo.

## Materials and Methods

### Cell Culture and Treatment

PIG1 (an immortalized human normal melanocyte cell line) and PIG3V (a human vitiligo melanocyte cell line) were kindly gifted from department of Dermatology, Xijing Hospital, Fourth Military Medical University. The human normal melanocytes were isolated from neonatal foreskin, and human vitiligo melanocytes were isolated from non-lesional skin of a vitiligo patient. These cells were immortalized by retroviral transfection with HPV16 E6 and E7 genes (Le Poole et al., 1997; Le Poole et al., 2000). PIG1 and PIG3V were cultured in Medium 254 (Gibco, Grand Island, NY) supplemented with human melanocyte growth supplement (HMGS) (Gibco), 10% fetal bovine serum (Biological Industries, Israel), and a penicillin-streptomycin antibiotic mix (Biological Industries, Israel) at 37°C in the presence of 5% CO2, as previously reported (Shi et al., 2016). Oxidative stress in PIG 1 or PIG 3Vwas induced by 1.0 mM H_2_O_2_ (Sigma-Aldrich, USA) for 24 h. PF (Sigma-Aldrich, USA) at the concentration of 50 µM were added 24 h before H_2_O_2_ stimulation. To determine whether *JNK* pathway play a role in PF-induced *Nrf2* activation, melanocytes were treated with 25 µM *JNK* inhibitor (SP600125) (MedChemExpress, USA).

### Determination of Cell Viability by MTS Assay

The MTS assay was determined by the Cell Titer 96AQUeous One Solution Cell Proliferation Assay (Promega, Madison, WI). In brief, cells were seeded into 96−well plates (1,500–2,000 cells/well) overnight, and then treated with PF at concentrations ranging from 50 µM to 400 µM for 0–3 days. Then MTS kit was applied and cells were incubated at 37°C for 2 h, absorbance was read using a microplate reader at 490 nm (BioTek, USA).

### Analysis of Cell Cycle by Flow Cytometry

Cell cycle was detected by PI/RNase staining buffer (BD Biosciences, USA). PIG1 and PIG3V were seeded in a 6-well plate (2-2.5×10^5^ cells/well) and treated with 50 µM PF for 24 h. After dealing with 1.0 mM H_2_O_2_ for another 24 h, cells were harvested and fixed with 70% ethanol over night at 4°C. Afterwards, cells were washed using PBS, incubated with propidium iodide (PI)/RNAase staining buffer at room temperature for 30 min and tested with BD LSRFortessa instrument (BD Biosciences, USA). Cell cycle was analyzed using ModFit LT (version 3.3; Verity Software House, Topshame, ME, USA).

### Apoptosis Analysis by Flow Cytometry

PIG1 and PIG3V were plated into 6-well plates at a density of 2-3×10^5^ cells/well. Cell apoptosis was detected by using Annexin V−fluorescein isothiocyanate (FITC)/propidium iodide (PI) staining (BD Biosciences, USA). In brief, after disposal by PF and H_2_O_2_ like cell cycle, cells were harvested and then washed with cold PBS. After centrifugation, cells were resuspended with Annexin V−FITC (5 µl) and PI (5 µl) for 15 min in the dark. A flow cytometer (BD Biosciences, USA) was used to analyze the samples.

### Measurement of ROS

The ROS levels were determined using the ROS assay kit (Beyotime, China). After drugs treatment, cells were harvested, and then washed with PBS and stained with 10µM 2',7'−dichlorodihydrofluorescein diacetate (DCFH-DA) for 30 min at room temperature in the dark. After washing with PBS, the samples were analyzed for the fluorescence of DCF with the BD LSRFortessa instrument (BD Biosciences, USA).

### Detection of SOD and CAT Activities

Cells were treated as described previously. Activities of anti-oxidative enzymes including CAT and SOD were respectively detected with Catalase Assay kit (Beyotime, China) and Total Superoxide Dismutase Assay kit with WST-8 (Beyotime, China), according to the manufacturer's protocols. Then, the SOD and CAT activities were normalized to protein levels.

### Immunofluorescence Staining

PIG1 and PIG3V were seeded and treated in 12-well plates. After being washed and fixed, cells were incubated with melanoma gp100 (Abcam, UK) antibody overnight at 4°C. Following three washes with PBS, cells were incubated with the goat antirabbit Alexa Fluor^®^ 594 (IgG H&L) antibody (Abcam, USA) for an additional 1h in darkness. After rinsing with PBS, Phalloidin (Cell Signaling Technology, USA) were added to the cells. Then cells were incubated for 30 min at room temperature, and nuclear dyed with DAPI (Solarbio, China) for 10 min at room temperature. Fluorescent images were obtained by the BioSpa Live Cell Analysis System (BioTek, USA).

### Western Blotting Analysis

PIG1 and PIG3V were disposed and then lysed in RIPA buffer containing 1% PMSF (Beyotime, China) and phosphatase inhibitor (Thermo Scientific, USA) for 10 min at 4°C. Cell lysates were centrifuged at 12000 × g for 10 min at 4°C, and the protein concentrations were precisely determined using the BCA Protein Assay Kit (Beyotime, China). Proteins (20 μg) stained with 5× loading buffer were boiled for 5 min, separated by 12% SDS-PAGE and then transferred onto polyvinylidene difluoride membranes (Merck Millipore; Germany). The membranes were blocked with 5% skim milk (Solarbio, China) for 2 h and incubated overnight at 4°C with primary antibodies as follows: total *Nrf2*, phospho-*Nrf2* (p-Nrf2), NAD(P)H: quinone reductase (*NQO1*) (Abcam, USA), total c-Jun amino terminal kinase (total *JNK*), phospho-*JNK*, *HO-1*, and *Tublin* (Cell Signaling Technology, USA). After washed extensively with TBST, the membranes were incubated with goat anti−rabbit or anti−mouse immunoglobulin G horseradish peroxide conjugated secondary antibodies (ZSGB-BIO, China) at 37°C for 1 h. Finally, the bands were assessed by the Bio-Imaging Systems (MF-ChemiBIS 2.0, Israel).

### Data Analysis

Data analysis was performed by GraphPad Prism version 7.0 software (GraphPad Software, San Diego, CA). One-way ANOVA was performed for multiple group comparisons and used the Sidak's multiple comparisons test for the comparison of two groups. Data was presented as mean ± SD (standard deviation) for at least three independent experiments. Adjusted *P*-value < 0.05 was considered statistically significant.

## Results

### PF Ameliorates H_2_O_2_-Induced Cytotoxicity in Melanocytes

**Figure 1:**
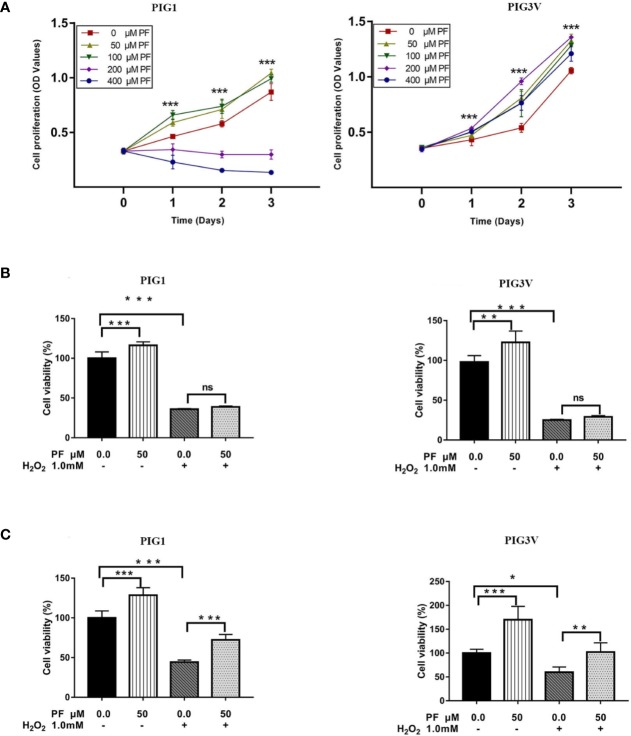
PF ameliorates H_2_O_2_-induced cytotoxicity in melanocytes. **(A)** Melanocytes were treated with different concentrations of PF for indicated times. Cell proliferation was determined by MTS assay. Melanocytes were pretreated with PF for 24 h **(B)** or 48h **(C)** and then exposed to 1.0 mM H_2_O_2_ for another 24 h. The cell viability was determined by MTS. All data were presented as the mean ± standard deviation across three independent experiments. *P < 0.05, **P < 0.01, ***P < 0.001, ns, not significant. H_2_O_2_, hydrogen peroxide; PF, Paeoniflorin; h, hours; M, mol/L.

We first evaluated the toxicity of PF on cell proliferation of PIG1 or PIG3V to select the dose for subsequent experiments. Concentration of 50 μM was chosen as it did not result in significant cytotoxicity in both PIG1 and PIG3V (**Figure 1A**). Treatment of melanocytes with 1.0 mM H_2_O_2_ for 24 h to induce oxidative stress as previous reports (Jian et al., 2016). The cell viability was significantly decreased after H_2_O_2_ treating for 24 h. Although pretreatment with PF for 24 h (**Figure 1B**) did not reverse the damage, when the treatment extended to 48 h (**Figure 1C**), it could significantly increase the cell viability in H_2_O_2_-induced melanocytes. Moreover, the cell viability was increased treated with PF compared with the control cells both 24 h and 48 h, Therefore, PF ameliorates H_2_O_2_-induced cytotoxicity in melanocytes.

### PF Regulating Cell Cycle Progression in G2/M Phase in Stressed Melanocytes

**Figure 2:**
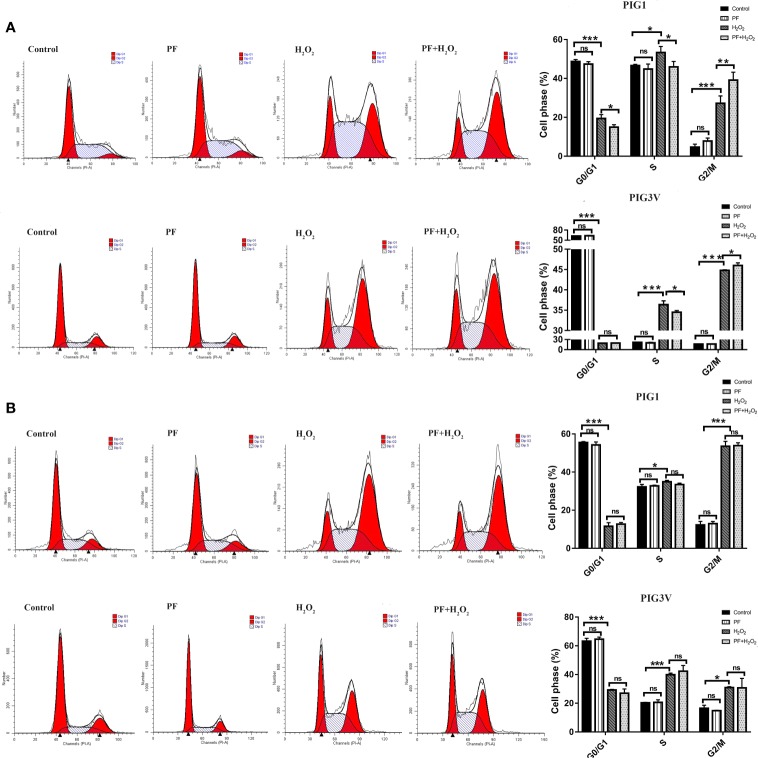
PF regulates cell cycle progression in G2/M phase in stressed melanocytes. Melanocytes were pretreated with PF for 24 h **(A)** or 48h **(B)** and exposed to 1.0 mM H_2_O_2_ for another 24 h. The cell cycles were analyzed by flow cytometry assay and the percentages of cells in each stage were shown as the right bar graphs. All data were presented as the mean ± standard deviation across three independent experiments. *P < 0.05, **P < 0.01, ***P < 0.001. ns, not significant; H_2_O_2_, hydrogen peroxide; PF, Paeoniflorin; h, hours; M, mol/L.

We investigated whether H_2_O_2_ influenced the cell cycle. After incubating with H_2_O_2_ for 24 h, PIG1 and PIG3V significantly increased at S phase and G2/M phase, with the fraction of G2/M phase increasing from about 5% to 27% in PIG1 and from 17% to 31% in PIG3V when compared to the control groups. Pretreatment with PF for 24 h caused the S phase decreased and the G2/M phase increased (**Figure 2A**), while 48 h did not (**Figure 2B**), in the stressed melanocytes, However, PF treated for 24 h or 48 h both had no influence on the cell cycle compared to the normal control cells. The results indicated that PF promoted cell cycle progression in G2/M phase to increase the cell proliferation in stressed cells.

### PF Ameliorates H_2_O_2_-Induced Apoptosis in Melanocytes

**Figure 3:**
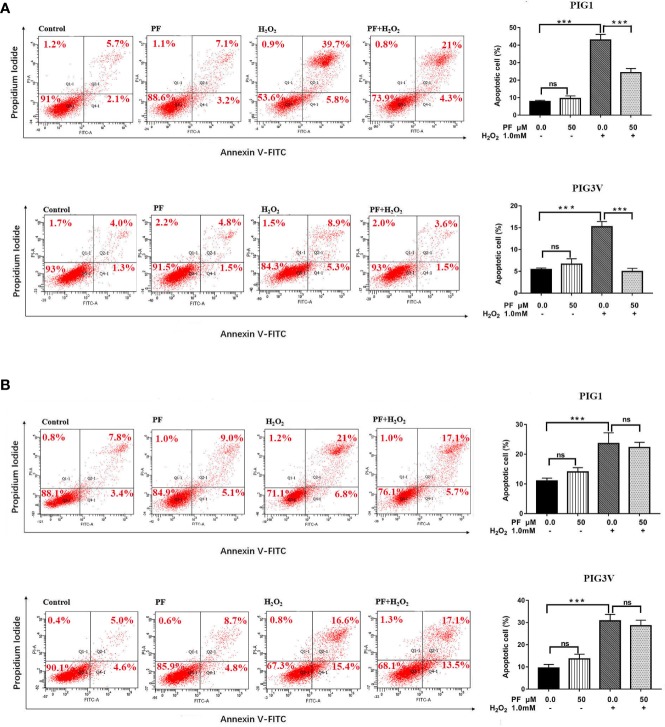
PF ameliorates H_2_O_2_-induced apoptosis in melanocytes. Melanocytes were treated with PF for 24 h **(A)** or 48h **(B)** and then treated with H_2_O_2_ for another 24 h. The cell apoptosis was analyzed by the flow cytometry and each figure shown was representative image from three independent experiments, the percentages of cells apoptosis were shown as the right bar graphs and the data were presented as the mean ± standard deviation across three independent experiments. ***P < 0.001. ns, not significant; H_2_O_2_, hydrogen peroxide; PF, Paeoniflorin; h, hours; M, mol/L.

We also investigated whether PF protects melanocytes from H_2_O_2_-induced cell death, flow cytometry analysis was used to quantify apoptosis in PIG1and PIG3V. After treatment with1.0 mM H_2_O_2_ for 24 h, the average percentage of apoptotic cells markedly increased to about 45% in PIG1 and 14% in PIG3V (**Figure 3A**). Pretreatment of PF for 24 h could attenuate apoptosis in both cell lines (**Figure 3A**), but 48 h not (**Figure 3B**). There were no significant differences in apoptosis rates in the PF groups when compared to the normal control group. These results suggested that PF can improve H_2_O_2_-induced cytotoxicity in melanocytes.

### PF Attenuates H_2_O_2_-Induced Oxidative Stress in Melanocytes

**Figure 4:**
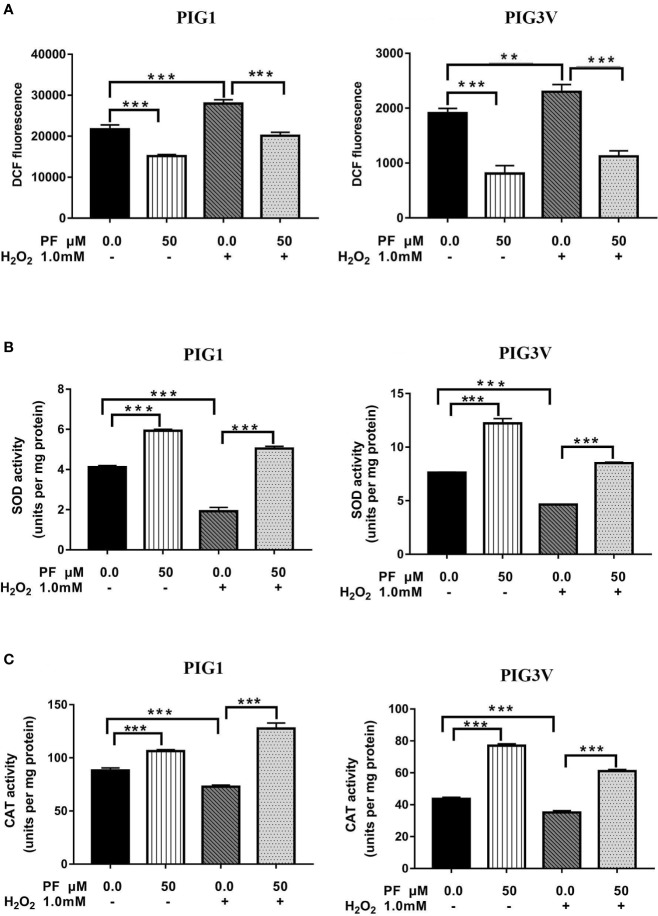
PF attenuates H_2_O_2_-induced oxidative stress in melanocytes. Melanocytes were pretreated with PF for 24 h and exposed to 1.0 mM H_2_O_2_ for another 24 h. **(A)** The ROS production were analyzed by the flow cytometry. The activities of antioxidant enzymes SOD **(B)** and CAT **(C)** were respectively detected with Catalase Assay kit and Total Superoxide Dismutase Assay kit with WST-8 assay. All data were presented as the mean ± standard deviation across three independent experiments. **P < 0.01, ***P < 0.001. ns, not significant; ROS, reactive oxygen species; SOD, superoxide dismutase; CAT, catalase; H_2_O_2_, hydrogen peroxide; PF, Paeoniflorin; h, hours; M, mol/L.

We pre-treated PIG1 or PIG3V with PF for 24 h and then challenged the cells with H_2_O_2_ for another 24 h. Afterwards, related markers involving in oxidant stress were detected. The specific marker of oxidant stress ROS significantly increased after H_2_O_2_ irritation for 24 h and PF could markedly reduce it (**Figure 4A**). The activities of antioxidant enzymes SOD and CAT significantly decreased in H_2_O_2_-induced cells, and PF pre-treatment for 24 h could abrogate the inhibition effects of oxidative stress (**Figures 4B, C**). The levels of ROS were decreased and the concentrations of SOD and CAT were increased after PF treated for 24 h compared to the control cells. Therefore, these results suggest that PF is able to reduce ROS and activate the antioxidant enzymes to potentiate melanocytes antioxidant capacities.

### PF Promotes Dendrite Formation and Melanosome Transport Under H_2_O_2_-Induced Oxidative Damage

**Figure 5:**
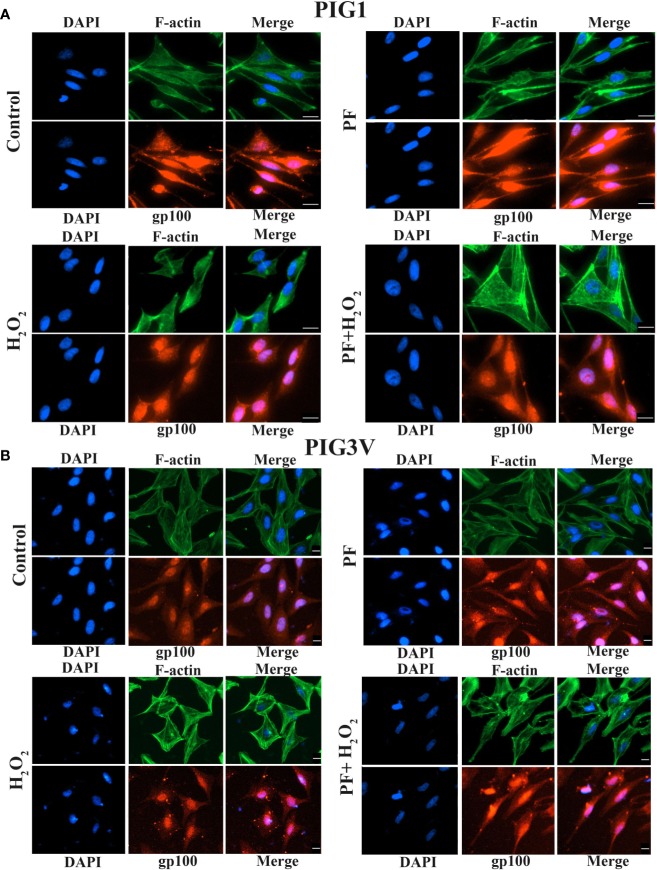
PF promotes dendrite formation and melanosome transport under H_2_O_2_-induced oxidative damage. Melanocytes were exposed to PF for 24 h and were further treated with H_2_O_2_ for another 24 h. The dendrite lengths were increased and contained more melanosomes in both stressed PIG1 **(A)** and PIG3V **(B)** when pre-treated with PF compared to H_2_O_2_ treated only. Then cells were fixed and stained with antibodies recognizing F-actin (phalloidin, green) and melanosome (gp100, red). Nuclei were counterstained with DAPI (blue). Immunofluorescence was used to determine the localization and expression of melanosomes and F-actin in the cells. Scalebar = 20µm. H_2_O_2_, hydrogen peroxide; PF, Paeoniflorin.

Actin cytoskeleton involved in the formation of new melanocyte dendrites as the process needs actin polymerization (Sit and Manser, 2011). Dual immunofluorescence of F-actin and melanosome was used to evaluate if PF influences the formation of dendrites and melanin synthesis. The dendrite lengths of PIG1 and PIG3V significant shortened and the melanosomes decreased when cells were treated with 1.0 mM H_2_O_2_, compared with untreated cells. Treating with PF can increase the length of dendrites and make melanosome scatter to the tips in stressed cells, the results showed that PF probably decreased the actin polymerization to partly increase the lengths of dendrites. Moreover, the results revealed that F-actin mainly distributed at the cell periphery and melanosomes mainly aggregated around the nucleus. Co-localization of F-actin with melanosomes near the nucleus region and dendrites of both PIG1 and PIG3V (**Figure 5**) which suggested that F-actin may not be a key regulator of melanosome trafficking. Taken together, the results indicated that PF may promote dendrite extension by inhibiting the actin polymerization and melanin synthesis.

### PF Activates *Nrf2/HO-1* Pathway in H_2_O_2_-Treated Melanocytes

**Figure 6:**
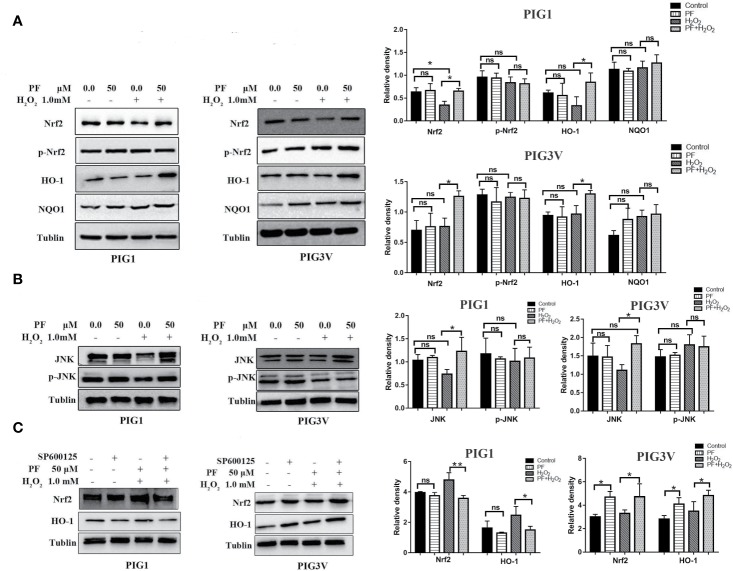
PF activates *JNK/Nrf2/HO-1* pathway in H_2_O_2_-treated melanocytes. Melanocytes were treated with PF for 24 h and further treated with H_2_O_2_ for another 24 h. **(A)** Western blots of total *Nrf2, p-Nrf2*, and its downstream antioxidant enzymes, *NQO1*, and *HO-1*. **(B)** The protein levels of *JNK* and *p-JNK*, **(C)** The levels of *Nrf2* and *HO-1* proteins in melanocytes treated with SP600125 and PF followed by H_2_O_2_. The quantitative analysis of *Nrf2, p-Nrf2, NQO1*, and *HO-1* **(A)**, *JNK* and *p-JNK* **(B)**, and *Nrf2* and *HO-1* **(C)** using Tublin as normalization were showed at the right, respectively, all data are presented as mean ± standard deviation. n = 3 per group, *P < 0.05, **P < 0.01, ns, not significant; H_2_O_2_, hydrogen peroxide; PF, Paeoniflorin; M, mol/L; h, hours; *JNK*, c-Jun N-terminal kinase. p-, phosphorylated.

*Nrf2* and its target genes which known as phase-II enzymes, are critical in regulating oxidative stress (Nguyen et al., 2009). To determine whether PF activates this signaling system in H_2_O_2_-induced oxidative stress, we assessed the levels of *Nrf2, p- Nrf2*, and its main downstream antioxidant enzymes, *NQO1*, and *HO-1* (**Figure 6A**). The results showed that pretreatment with PF can significantly augmented *Nrf2* and *HO-1* expressions, but not *p-Nrf2* and *NQO1*, in H_2_O_2_-induced PIG1 or PIG3V, compared to H_2_O_2_ only groups. Besides, PF treatment did not result in different expressions of Nrf2, p- Nrf2, NQO1 and HO-1 when compared to control group. Moreover, the *Nrf2* expression had a significant reduction when cells were treated with only H_2_O_2_, compared to control group in PIG1. These results suggest that PF possibly activated the *Nrf2/HO-1* pathway to counteract H_2_O_2_-induced oxidative damage in PIG1 and PIG3V.

### *JNK/Nrf2* Signaling Mediates Antioxidative Effects of PF on H_2_O_2_-Induced Melanocytes

Previous studies showed that *JNK* is an important upstream regulator of *Nrf2* (Xu et al., 2006; Yao et al., 2007). Thus, we further studied the ability of PF to upregulate the *JNK*, and *p-JNK*. We found that PF potentiated *JNK* under oxidative stress compared to H_2_O_2_ alone group (**Figure 6B**). Cells treated with SP600125 (*JNK* inhibitor) can decrease *Nrf2* expression in PIG1, but increased the expression of *Nrf2* in PIG3V (**Figure 6C**) (*P* < 0.05). Therefore, PF exerts its antioxidative effects may through *JNK/Nrf2/HO-1* signaling pathway in H_2_O_2_-treated melanocytes.

## Discussion

**Figure 7:**
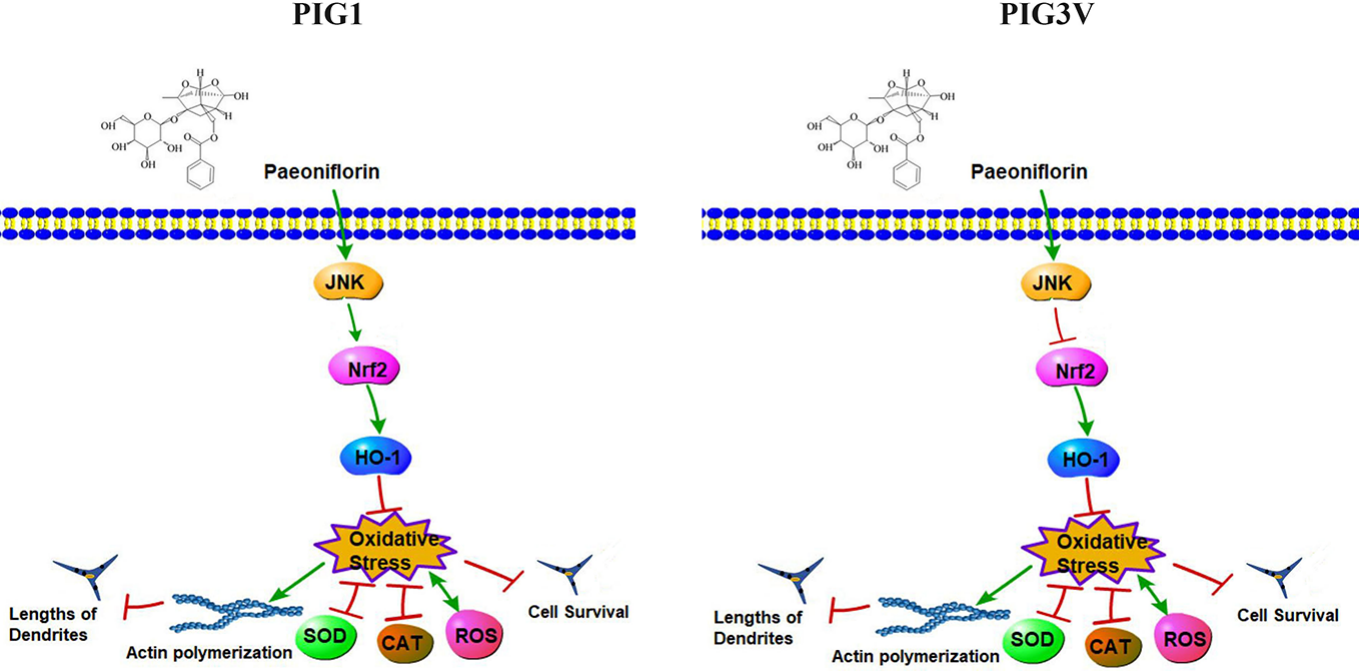
The signaling pathway network by which PF resists H_2_O_2_-induced oxidative stress in melanocytes. PF may depended on the *JNK/Nrf2/HO-1* pathway to reduce oxidative damage which was reflected in suppressing reactive oxygen species (ROS) accumulation and enhancing the productions of superoxide dismutase (SOD) and antioxidant enzymes catalase (CAT), therefore, PF inhibited cell apoptosis and promoted cell proliferation., Moreover, PF promoted dendrite extension probably by inhibiting the actin polymerization in stressed cells. In addition, *JNK* increased the *Nrf2* expression in PF-pretreated PIG1, but inhibited the expression of *Nrf2* in PF-pretreated PIG3V under oxidative stress. Pathway Builder Tool 2.0 was used to draw this picture.

The present study found PF at 50 µM significantly increased cell viability, decreased ROS and enhanced antioxidant enzymes SOD and CAT in stressed cells. Furthermore, we found that PF resulted in significant increase of endogenous anti-oxidant *JNK*, *Nrf2*, and *HO-1* in stressed cells. Inhibition of *JNK* can markedly impaired the expression of *Nrf2* in PIG1, but increased it in PIG3V (**Figure 7**). Previous studies have shown that upregulation of *Nrf2/HO-1* could increase the capability to cope with H_2_O_2_-induced stress damage in PIG3V (Jian et al., 2014). Our results were consistent with previous findings and showed the functional activation of antioxidant signaling pathway by PF.

Oxidative damage can result in the development of vitiligo and deleterious effects, ROS can lead to apoptosis of both melanocytes and keratinocytes in vitiligo epidermis (Bellei et al., 2013; Yang et al., 2017). Pradhan et al. found the level of ROS was significantly elevated in vitiligo patients when compared to healthy controls (Pradhan et al., 2014). It has been reported that TGP could promote re-pigmentation in vitiligo patients by improving the CD4+/CD8+ T lymphocyte ratio and the CD4+CD25+ Treg level in peripheral blood (Ye et al., 2013; Shen et al., 2019). A recent study demonstrated that PF can enhance melanin synthesis by the extracellular signal-regulated kinase (*ERK*)/cAMP-response element binding (CREB) pathway with up-regulation of microphthalmia-associated transcription factor (*MITF*) and tyrosinase-related protein 1 (*TRP-1*) (Hu et al., 2020). In addition to the immunomodulatory and melanin synthesis effects (Ye et al., 2013; Hu et al., 2020). Our study showed that PF can suppress ROS accumulation and other oxidative markers in melanocytes which suggested that PF possesses anti−oxidative capacity and may be a potential medicine of vitiligo treatment.

Melanocytes had the ability to form dendrites and by which melanosomes can be transported to surrounding keratinocytes to contribute to skin darkening. The length and numbers of melanocyte dendrites will markedly influence the process of melanosomes transport. (Sulaimon and Kitchell, 2003; Yamaguchi et al., 2007). Our immunofluorescence analysis confirmed that melanocyte dendrites were elongated and melanosomes are distributed around the nucleus and the tips when stressed cells were pretreated with PF. When combining these immunofluorescence results with pathway results, it suggests that PF may inhibit the actin polymerization to induces dendrite elongation, Moreover, PF promotes dendrite formation and melanin synthesis probably by increasing the *JNK/Nrf2/HO-1* pathway in both cell lines.

Previous study showed that under oxidative stress, *Nrf2* will dissociates from Keap1, and binds to ARE to activate the expression of *HO-1* and *NQO1*. Defective *Nrf2* activation in melanocytes involved in the onset of vitiligo (Kobayashi and Yamamoto, 2005; Jian et al., 2014). We found that PF increased the *Nrf2/HO-1* expression in H_2_O_2_-induced melanocytes to reduce the oxidative damage. The *Nrf2* expression can be enhanced by *JNK* in PIG1, in contrast, be reduced by *JNK* in PIG3V which may cause insufficient activation of *Nrf2-ARE* pathway to coping with oxidative stress.

In conclusion, we determined that PF may activate the *JNK/Nrf2/HO-1* pathway to protect melanocytes from oxidative damage. The effective concentration of paeoniflorin in the epidermis of vitiligo patient needs further study, as finding the optimal minimal doses of PF can reduce the potential risk of adverse effects and decrease patient's economic burden. Additional studies are needed to further confirm the ability of PF in the treatment of vitiligo.

## Data Availability Statement

All datasets generated for this study are included in the article/supplementary material.

## Author Contributions

Designed and performed the article: JY. Data analysis: JY, YL, SJ, YF, HW, and YW. RQ and H-DC participated in revising the manuscript. Provide approval for publication of the content: YW, RQ, and H-DC. All authors contributed to manuscript revision, and read and approved the submitted version.

## Funding

This work was supported by the National Natural Science Fund (Grant number 81972940) and Liaoning Province Natural Science Fund (Grant number 2019-ZD-0763)

## Conflict of Interest

The authors declare that the research was conducted in the absence of any commercial or financial relationships that could be construed as a potential conflict of interest.
